# Development and implementation of a worksite-based intervention to improve mothers’ knowledge, attitudes, and skills in sharing information with their adolescent daughters on preventing sexual violence: lessons learned in a developing setting, Sri Lanka

**DOI:** 10.1186/s12889-024-18416-x

**Published:** 2024-04-08

**Authors:** Dilini Mataraarachchi, P.K. Buddhika Mahesh, T.E.A. Pathirana, P.V.S.C. Vithana

**Affiliations:** 1 Family Health Bureau, Colombo, Sri Lanka; 2 Provincial Director of Health Services, Colombo, Sri Lanka; 3 Base Hospital, Panadura, Sri Lanka

**Keywords:** Adolescent sexual health, Sexual violence, Family-based sexuality education

## Abstract

**Background:**

Sexual violence among adolescents has become a major public health concern in Sri Lanka. Lack of sexual awareness is a major reason for adverse sexual health outcomes among adolescents in Sri Lanka. This study was intended to explore the effectiveness of a worksite-based parent-targeted intervention to improve mothers’ knowledge, and attitudes on preventing sexual violence among their adolescent female offspring and to improve mother-daughter communication of sexual violence prevention with the family.

**Methods:**

“My mother is my best friend” is an intervention designed based on previous research and behavioral theories, to help parents to improve their sexual communication skills with their adolescent daughters. A quasi-experimental study was conducted from August 2020 to March 2023 in randomly selected two Medical Officer of Health (MOH)areas in Kalutara district, Sri Lanka. Pre and post-assessments were conducted among a sample of 135 mothers of adolescent girls aged 14–19 years in both intervention and control areas.

**Results:**

Out of the 135 mothers who participated in the baseline survey, 127 mothers (94.1%) from the intervention area (IA) physically participated in at least one session of the intervention. The worksite-based intervention was effective in improving mothers’ knowledge about adolescent sexual abuse prevention (Difference in percentage difference of pre and post intervention scores in IA and CA = 4.3%, *p* = 0.004), mother’s attitudes in communicating sexual abuse prevention with adolescent girls (Difference in percentage difference of pre and post intervention scores in IA and CA = 5.9%, *p* = 0.005), and the content of mother-daughter sexual communication (Difference in percentage difference of pre and post intervention scores in IA and CA = 27.1%, *p* < 0.001).

**Conclusions and recommendations:**

Worksite-based parenting program was effective in improving mothers’ knowledge about sexual abuse prevention among adolescent daughters and in improving the content of mother-daughter communication about sexual abuse prevention. Developing appropriate sexual health programs for mothers of different ethnicities, and cultures using different settings is important. Conduction of need assessment programs to identify the different needs of mothers is recommended.

**Supplementary Information:**

The online version contains supplementary material available at 10.1186/s12889-024-18416-x.

## Background

Sexual violence and coercion involving children are seen in every part of society occurring in homes, workplaces, communities, and public spaces. World Health Organization defines sexual violence as “any unwanted sexual act, comment or advance encompassing various forms of abuse and exploitation” [1].

The UNICEF reports depict that worldwide, at least one in ten girls under the age of twenty are being forced into some form of sexual activity [2]. However, cases of sexual abuse and violence among children and adolescents in Sri Lanka are often under-reported due to stigma and ignorance [3]. A study reported that 14% of both male and female students in Sri Lanka suffered some form of sexual abuse [4]. A significant 23% of sexually active school adolescents experienced non-consensual sexual intercourse [5].

The literature shows that lack of awareness and poor life skills are the root causes of the higher incidence of sexual abuse among adolescents [6]. Despite more than three decades of school sexual health education, Sri Lankan adolescents lack adequate sexual health knowledge and receive conflicting messages [7].

Global research advocates family-based sexuality education, as this will create an open and comfortable environment for discussion about sexuality, relationships, and other related topics [8, 9]. Family-based sex education enables children to have early access to quality information on sexual matters, develop healthy attitudes towards sex and relationships, reduce risky behaviors, and make informed decisions about their sexual health.

In a family, the role of the mother is more pronounced and can have an impact on the health of other members [10]. Literature indicates that well-informed and prepared mothers are one of the best sources of sexual health information for adolescent girls [11].

In Sri Lanka, the stigma around the topic, coupled with various religious and mythological beliefs, discourages the implementation of school or community-based sex education for children. Nevertheless, the ability to tailor sexual health messages to suit one’s family values, religious beliefs highlights family-based sex education as a suitable approach for South Asian settings like Sri Lanka [12]. However, parental discomfort and lack of awareness of adolescent sexual health and lack of skills on sexual health communication, pose significant challenges hindering the opportunity to family based sexual education in the country [12].

It was discovered that 67% of Sri Lankan parents lack knowledge about child sexual abuse [13].The objective of this study was to develop a comprehensive intervention for mothers of adolescent girls, to enhance their understanding and enable them to convey crucial information to their daughters about preventing child sexual. The study aimed to evaluate the potential for family-based sex education in the study context. This is the first ever study to explore the potential for family-based sex education in this study context.

## Methods

### Setting for the intervention

Intervention was carried out in seven randomly selected government work-sites in IA and CA in Kalutara district. A spacious room or the auditorium in the work place was chosen as the setting for the intervention.

### Study period

The intervention was conducted in August 2020 while the baseline assessment was carried out one week prior to the intervention. Post-interventional assessment was carried out in March 2021.

### Target audience

Target audience for the intervention was mothers of adolescent girls aged 14–19 years.

### Planning of the intervention

The intervention development process involved technical experts, policymakers, practitioners, and representatives from the target population [14]. The intervention was based on the views and concerns of its users. Results of a descriptive-cross sectional study that was conducted among the adolescents in the study setting [15] and findings of a qualitative study carried out among mothers of adolescent girls to explore their views on providing sexual health information to their children [12] were taken into account during the intervention development.

The intervention in the present study was inspired by the information-motivation and behavioral skills model. Further, previous literature on similar parenting interventions to improve mothers’ knowledge, attitudes, and communication skills on adolescent sexual health matters was looked into.

### Piloting the intervention

The intervention was piloted at a government work site in Colombo, involving fifteen eligible mothers. Two public health specialists and an adolescent psychiatrist supervised the sessions. Practical issues regarding the timing of each session and logistics were identified. Participant feedback was collected to pinpoint areas needing improvement. The intervention content, lecture timing, and interactive sessions were adjusted based on participant suggestions. Handbooks and materials were distributed, with follow-ups conducted after two weeks to assess participant’s engagement. Mothers were inquired about the material readability and clarity. Post-pilot experts discussed identified issues and made necessary corrections.

### Intervention implementation

“My mother is my best friend” was carried out as two sessions one week apart, followed by a one-hour follow-up session six weeks apart. Trainer guide and all materials to be used during the session and material to be distributed among mothers following the session were developed with the expert opinion, while content and consensus generation among the experts on the content of the program was done using the Modified Delphi technique using electronic mail service.

The take-home materials were left at the workplace for the mothers who could not participate in the second session of the intervention. An online training program was carried out for the mothers who missed the sessions intervention. The online program was conducted in two separate sessions and the mothers who missed at least one session of the intervention were invited to participate.

### Program content

The intervention for the mothers included;

- Activity I - A short lecture presentation on physiological changes during adolescence.
- Activity II - A short lecture presentation on parent guide to adolescent sexual violence prevention.
- Activity III - A lecture presentation on how to communicate with your teen about protecting herself from sexual violence.
- Activity IV - Two video presentations that showed how to catch up with teachable moments and initiate a sexual conversation with an adolescent girl, and the techniques that can be used when responding to difficult questions forwarded during such discussion.
- Activity V - A handbook on preventing sexual violence among adolescent girls, to refer to after the program.
- Activity VI - Case scenarios for role play.
  All participant mothers were allocated into three small groups. Each group was provided with a case scenario. Each group was asked to narrate a brief role-play between a mother and a daughter about the given case scenario. Feedback from the audience was obtained after each role-play.
- Activity VII - Table topics–
  The table topics consisted of some questions, including general questions and a few sex and relationship-related questions. Mothers were expected to discuss each of the topics randomly with the children as a family game.
- Activity VIII - Checklist–
  A checklist was developed and given to all mothers for the self-assessment of the level of communication with adolescent daughters on preventing sexual violence. The checklist was assessed by the PI during the follow-up session, six weeks following the intervention.

#### Implementation fidelity of the intervention

All programs were facilitated by the principal investigator and one other medical officer, who had special training in adolescent health and experience in working with adolescents and their parents. The facilitators adhered to the trainer’s guide developed with expert opinion, to preserve the uniformity of the program when conducting each session.

### Monitoring of the intervention

Three months following the implementation of the intervention, the PI carried out an online follow-up session with the mother participants where they were inquired about the progress of the communication with daughters and for any problems they had encountered while practicing communication at home. Those who were not available at the online follow-up session were followed up over the phone.

**Control area**- Intervention was not implemented in the control area until the post-interventional assessment was done. Following the post-interventional survey, one hour lecture on sexual violence prevention among adolescent girls was carried out with the distribution of IEC material.

### Evaluation of the effectiveness of the intervention

#### Study design

Quasi experimental study design was used.

### Sample size calculation

Pre and post-interventional evaluation of the intervention was carried out among a sample of 135 mothers working in selected government worksites in both IA and CA. Pocock’s formula, a standard formula for sample size calculation of intervention studies was used to determine the sample size [16].

### Inclusion criteria

Eligible participant were Sinhala mothers having an adolescent daughter aged 14–19 years, and is living with the daughter in the same household for at least two days per week.

### Exclusion criteria

The following groups were excluded from the pre and post intervenion surveys, although they were allowed to participate in the intervention.

- Mothers diagosed with any severe mental disability at the time of the intervention would prevent them from effectively engaging with the daughter.
- Mothers with adolescent daughters who had cognitive or communication disabilities that would prevent her from effectively engaging with her mother.

### Sampling procedure

Out of the 13 MOH areas in Kalutara district, two MOH areas that share similar socio-demographic characteristics were selected as IA and CA. A list of government working places with more than fifty female employees in both IA and CA were taken from the relevant Divisional Secretariat office. From each list 9 government working places were randomly selected. From each worksite fifteen female employees meeting the eligibility criteria were included in to the study.

### Data collection

Data collectors visited the worksite one week prior to the intervention and six months after the implementation of the worksite intervention. Data was collected using an interviewer administered questionnaire. Trained interviewers made pre and post interventional visits to the worksites to collect data from study participants.

### Data Analysis

All data were coded and entered into a database using a standard statistical package (SPSS 25). Data cleaning and checking were done by the PI. Socio-demographic information of the participants was presented in numbers and percentages.

The evaluation of the effectiveness of the intervention was carried out in 3 stages;

1. Comparison of pre-interventional scores between intervention and control areas.
2. Comparison of post-interventional scores between intervention and control areas.
3. Comparison of pre and post interventional scores within intervention and control area.

The total percentage scores were calculated for each subscale. Since the total percentage scores were not normally distributed (Shapiro-Wilk test *p* < 0.05, Kolmogov-Smirnov test < 0.05), the analysis was carried-out using non-parametric tests. Between area comparison of knowledge, attitudes and communication practices of the mothers was carried out using Mann- Whitney U test, while Pre and post-test comparison within IA and CA area was done using Wilcoxon Signed Rank test.

The effect sizes were computed to determine the strength of the association. Since the outcomes were not normally distributed, the effect sizes were calculated using non-parametric effect size estimator, Cohen’s r.

Interpretation of effect size.

Effect size < 0.1 = no effect

Effect size 0.1–0.3 = Small effect

Effect size 0.3 = 0.5 = Medium effect

Effect size > 0.5 = Large effect

The percentage difference in pre and post intervention scores in IA and CA was calculated. Percentage Increase = [(Post-Intervention Score - Pre-Intervention Score) / Pre-Intervention Score] * 100

## Results

**Fig. 1:**
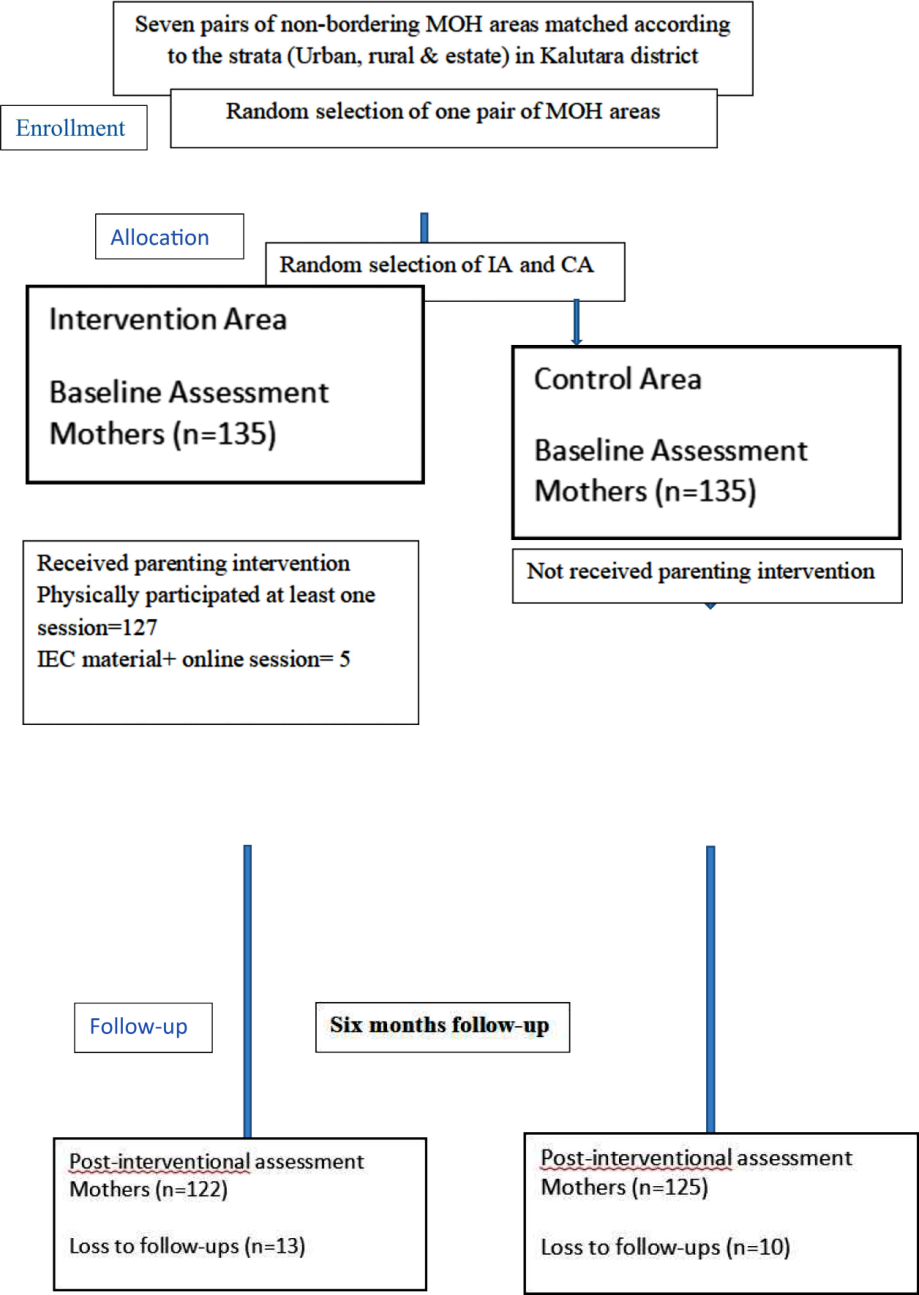
CONSORT diagram graphically describes the participant’s disposition throughout the study

The response rate for the pre-intervention was 100% for the mothers in both IA and CA. Out of the mothers who participated in the baseline survey 127 mothers (94.1%) from the intervention area (IA), physically participated in at least one session of the intervention. Five out of eight participants who missed both sessions participated in the online session. Participants who did not participate in the intervention (*n* = 3) were considered lost-to-follow-ups (Fig. 1).

For the post-interventional assessment, 122 mothers from the IA, and 125 mothers from the Control area (CA) participated giving a final response rate of 90.3% (122/135) and 92.6% (125/135) respectively.

The reasons for loss-to-follow-up among IA mothers were later change of mind (*n* = 6),

the husband was against participating in the study (*n* = 4), and change of workplaces during the six months follow-up period (*n* = 3). Among the CA mothers, five changed their minds later in the course, while four mothers said their husbands were against the idea. One mother had shifted workplaces due to a change of residence.

Out of all 135 mothers recruited to the study in IA and CA, who participated in the baseline assessment, the number of mothers who responded to the six months post-interventional survey was 122 and 125 in IA and CA, respectively. Therefore, the loss to follow-up rate calculated was 9.6% (*n* = 13) for the mothers in IA and 7.4% (*n* = 10) for the mothers in CA (Fig. 1).

At 95% confidence level, there was no significant difference between the participants who completed the study and the drop-outs, in their age, educational status, civil status, or the age of the adolescent daughter, which suggested random dropouts (Table 1).

**Table 1:** Comparison of the basic demographic characteristics of the participants who completed the study with the lost-to-follow ups

Demographic Characteristic	Respondents		Dropouts		Significance
	N	%	N	%	
**Age (n = 269)**					
30–40 years	22	8.9	2	8.3	X^2^ = 0.34, Df = 2
40–50 years	178	72.4	17	75.0	*P* = 0.85
50–60 years	46	18.7	4	16.7	
**Religion (n = 270)**					
Buddhist	234	94.8	22	97.0	X^2^ = 0.85, Df = 1
Non-Buddhist	13	5.2	1	3.0	*P* = 0.3
**Educational status (n = 268)**					
Below O/L	12	4.9	0	0.0	Fisher’s Exact
O/L and above	233	95.1	23	100.0	*P* = 0.6
**Civil status (n = 270)**					
Married	235	95.1	20	87.5	Fisher’s Exact
Unmarried	12	4.9	3	12.5	*P* = 0.13
**Daughters’ age (n = 270)**					
Less than 15	93	37.7	6	26.1	X^2^ = 1.34, Df = 2
15 years or more	154	62.3	17	73.9	*P* = 0.5

*Tests used for statistical analysis: Chi-square test and fisher’s exact test

### Preliminary effects of the intervention

There was no significant difference between the IA and CA mothers in their knowledge, attitudes about adolescent sexual violence prevention or in the content and frequency of communication with their daughters on sexual violence prevention (Table 2).

**Table 2:** Comparison of baseline knowldege, attitudes, content and frequency of communication practices of the mothers on preventing sexual violence in intervention and control areas

Component	Pre-intervention		Z Value	Significance	Effect size (r)
	IA Median IQR (*n* = 135)	CA Median IQR (*n* = 135)			
Knowledge about preventing sexual violence	86.4 (75.8–92.7)	87.3 (77.0-91.9)	-0.11	U = 9044 *P* = 0.91	0.006
Attitudes on communicating sexual matters with adolescent girls	70.8 (62.5–83.3)	75.0 (66.7–83.3)	-0.66	U = 8688 *P* = 0.51	0.04
Content of mother-daughter communication on sexual violence	75.0 (37.5–87.5)	62.5 (62.5–87.5)	-0.29	U = 8861 *P* = 0.77	0.04
Frequency of mother-adolescent daughter communication on sexual violence prevention	58.3 (41.7–75.0)	70.8 (50.0–83.0)	-2.38	**U = 7585** *P* = 0.02	0.14

*Statistical test used for the analysis is Mann-Whitney U test

According to the results mothers’ post-intervention knowledge, content, and frequency of communication with their daughters on sexual violence prevention was significantly higher compared to the baseline in the IA. In the IA the strength of association for mother’s knowledge improvement (0.3) and the content of communication (0.47) was moderate, while it was high for the frequency of communication. However, no such improvement could be seen in the control area. (Table 3).

**Table 3:** Comparison of pre and post intervention scores within intervention and control areas

Component	Area	Pre-intervention Median IQR	Post-intervention Median IQR	Z value	Significance	Effect size (r)
Knowledge for preventing sexual violence among girls	IA	86.1 (70.9–92.3)	89.6 (79.5–94.3)	-3.58	*P* < 0.01	**0.30**
	CA	87.3 (77.0-91.9)	89.6 (79.5–94.3)	-1.28	*P* = 0.2	0.11
Attitudes on communicating sex-related matters with adolescent girls	IA	70.8 (62.5–83.3)	75.0 (70.0–85.0)	-1.98	*P* = 0.06	0.17
	CA	75.0 (66.5–83.3)	75.0 (70.0–85.0)	-0.58	*P* = 0.56	0.05
Content of mother-daughter communication of sexual violence prevention	IA	75.0 (37.5–87.5)	87.5 (62.5–87.5)	-5.55	*P* < 0.01	**0.47**
	CA	62.5 (62.5–87.5)	70.0 (62.5–83.5)	-1.10	*P* = 0.27	0.09
Frequency of mother-daughter communication on sexual violence prevention	IA	58.3 (41.7–75.0)	70.8 (62.0-83.3)	-8.07	*P* < 0.01	**0.73**
	CA	70.8 (50.0–83.0)	66.7 (50.0-83.3)	-0.24	*P* = 0.81	0.02

*Statistical test used for analysis: wilcoxon-signed-rank test

The percentage increase in scores for mother’s knowledge, attitudes and communication on adolescent sexual abuse prevention pre and post intervention in IA and CA is shown in Table 4.

**Table 4:** Percentage difference in pre and post intervention scores in IA and CA

Component	Area	Pre-intervention Median IQR	Post-intervention Median IQR	Percentage difference in pre and post scores	Difference in percentage difference of IA & CA	Significance
Knowledge for preventing sexual violence among girls	IA	86.4 (75.8–92.7)	89.6 (79.5–94.3)	3.7%	4.3%	Z = 2.86
	CA	87.3 (77.0-91.9)	86.1 (72.2–92.3)	-0.6%		*P* = 0.004
Attitudes on communicating sex-related matters with adolescent girls	IA	70.8 (62.5–83.3)	75.0 (70.0–85.0)	5.9%	5.9%	Z = 2.75
	CA	75.0 (66.5–83.3)	75.0 (70.0–85.0)	0%		*P* = 0.005
Content of mother-daughter communication of sexual violence prevention	IA	75.0 (37.5–87.5)	87.5 (62.5–87.5)	16.6%	4.6%	Z = 1.03
	CA	62.5 (62.5–87.5)	70.0 (62.5–83.5)	12%		*P* = 0.30
Frequency of mother-daughter communication on sexual violence prevention	IA	58.3 (41.7–75.0)	70.8 (62.0-83.3)	21.4%	27.1%	Z = 8.24
	CA	70.8 (50.0–83.0)	66.7 (50.0-83.3)	-5.7%		*P* < 0.001

Comparison of post-interventional scores between IA and CA indicated that there is a significant difference in the mother’s knowledge about preventing sexual violence in adolescent girls and in the content of mother-daughter communication on sexual violence prevention. However, no significant difference was observed in mother’s attitudes or in the frequency of communication (Table 5).

**Table 5:** Post-interventional comparison of mothers’ attitudes, content and frequency of communication practices with daughters to prevent sexual violence between IA and CA

Component	Post-intervention		Z value	Significance	Effect size (r)
	IA Median IQR (n = 122)	CA Median IQR (n = 125)			
Mother’s knowledge about preventing sexual violence among adolescent girls	89.6 (79.5–94.3)	86.1 (72.2–92.3)	-2.94	U = 7111 *P* = 0.003	0.18
Attitudes on communicating sexual violence prevention with adolescent girls	75.0 (70.0–85.0)	75.0 (70.0–85.0)	-0.64	U = 8511 *P* = 0.52	0.04
Content of mother-daughter communication on sexual violence prevention	87.5 (62.5–87.5)	75.0 (62.5–87.5)	-2.88	U = 7123 *P* < 0.01	0.17
Frequency of mother-daughter communication on sexual violence prevention	70.8 (61.0-83.3)	66.7 (50.0-83.3)	-1.863	U = 7498 *P* = 0.06	0.11

*Statistical test used for the analysis is Mann-Whitney U test

## Discussion

The intervention in this study was inspired by the information-motivation and behavioral skills model, a framework that has consistently demonstrated empirical support in the sexual and reproductive health risk reduction in various key populations including adolescents and youth [17, 18]. The program structure was adapted from ‘Talking parents-healthy teens’, a successful worksite-based parenting program conducted in the US, which significantly increased parent-teen sexual health [19].

The intervention primarily focused on preventing sexual violence among adolescent girls. This emphasis stemmed from the previous research conducted among mothers of adolescent girls in the study setting, which revealed that mothers were more concerned about protecting their daughters from sexual violence [12, 20]. To enhance engagement and effectiveness, the intervention incorporated interactive sessions such as role-plays, video presentations, and take-home activities as suggested by previous literature [21–23]. Additionally, interactive games were incorporated to enhance easy communication between mothers and adolescents about SRH matters [23].

The study recognized the efficacy of utilizing worksite settings as platforms to implement health programs for working mothers. The support received from the worksite administration in conducting the program affirmed the potential to expand health promoting initiatives at worksites, moving beyond routine health assessments. Unlike challenges reported in previous literature concerning recruiting and retaining parents in parenting interventions [24], the work setting in this study facilitated the parent participation without additional effort. The enthusiasm of the worksite management to implement the program was partly driven by the growing number of sexual violence cases in the study setting and their interest in employee-assistance programs as work-life balance initiatives. Similar health-promoting interventions such as weight reduction programs or smoking cessation programs had been proven effective when conducted in worksite setting [25]. Moreover, evidence suggests that interventions aimed at facilitating parent-child sexual communication and reducing sexual risk behaviors among adolescents, conducted at parent’s workplaces, can be effective [26]. For instance, Talking Parents-Healthy Teens, a parenting intervention conducted at thirteen worksites in Southern California led to significant positive outcomes [27].

The study revealed a significant improvement in mother’s knowledge about preventing adolescent sexual abuse and in the content and frequency of sexual communication with their daughters among IA mothers six months following the intervention compared to baseline. However, when comparing areas, the intervention proved more effective in improving mother’s knowledge about preventing adolescent sexual abuse and the content of mother-daughter sexual communication among IA mothers compared to CA but no significant difference was observed in the frequency of communication. This disparity could be attributed to the fact that CA mothers reported higher frequency of sexual health communication with their daughters even at the baseline compared to the IA, which can be due to some unknown external factors. The finding aligns with a US study, “Talking parent-healthy teens”, where intervention area mothers reported more discussion of new topics (*p* < 0.001) and more frequency of conversations about sex compared to the control group [27].

Although there was no significant difference between the pre and post interventional scores for mother’s attitudinal change in IA, the percentage score increase in IA was significantly higher compared to the CA. A review of studies that have been carried out between 1980 and 2010 to evaluate the effectiveness of parenting interventions to improve sex communication indicated that very few interventions successfully influenced mothers’ attitudes [28]. Adopting different socio-psychological approaches would be essential to unfreeze mothers’ attitudes, which is crucial for ensuing the long-term sustainability of the intervention’s impact.

### Public Health implications of the study findings

According to the present study findings, parent-targeted interventions are an effective way of delivering sexual and reproductive health information to adolescents in Sri Lanka. The present intervention could be adopted with necessary modifications to the existing public health system to reduce sexual abuse among adolescent girls in the country.

### Strengths and limitations

The parenting intervention was developed considering the views of both the mothers and daughters in the same study setting, giving a more comprehensive approach to the study problem. The quasi-experimental study design enabled identifying the actual effect of the parenting intervention on the mothers and adolescent girls. The use of government worksites as the setting for the study, was an advantage since as it improved parent participation, reducing attrition bias. Carrying out face-to-face intervention was a challenge due to the COVID-19 pandemic situation in the country. Hence, we were unable to gain the full effect of a face-to-face intervention and follow-up sessions.

Furthermore, the pandemic resulted in both the mothers and daughters to work from home and spend more time together than usual. This may limit the generalization of the findings to the population during the COVID-19 free times.

### Conclusions and recommendations

The study highlights the effectiveness of mother targeted interventions in enhancing mothers’ knowledge and communication with their adolescent daughters regarding sexual health. Mothers’ enthusiasm to learn about adolescent health, indicated the necessity for future parent-targeted programs. The success of worksite setting in engaging mothers suggests its effectiveness for parenting programs.

The study calls for policymakers to recognize parents as a primary source of sexual health information for adolescent girls. It recommends implementing parent awareness and skill-building sessions alongside the existing school-based sexual health education programs. Additionally, education sector is encouraged to conduct such sessions in parallel to the current school sexuality education, promoting mothers’ role as early sexual health educators for their children even before adolescence.

### Future research

Future research should assess the effectiveness of a parenting intervention in improving mother-daughter sexual communication among unemployed mothers, considering potential difference between employed and unemployed groups. Additionally, it is crucial to include women who are employed in the private sector in these studies.

Furthermore, there is a need to develop interventions targeting fathers. The present intervention mainly focused on communicating sexual violence prevention with adolescents. Future interventions should encourage parent-adolescent communication on various sexual health topics.

The present intervention was implemented solely at large government worksites. Research should be conducted to evaluate the intervention’s effectiveness in small workplaces and private sector work settings.

### Electronic supplementary material

Below is the link to the electronic supplementary material.

Supplementary Material 1

## Data Availability

Data presented in this study are available with the corresponding author and can be produced on request.

## References

[1] Jewkes R, Dartnall E. Sexual violence. Int Encycl Public Heal. 2016;491–8.

[2] UNICEF. Sexual violence against children [Internet]. 2021. Available from: https://www.unicef.org/protection/sexual-violence-against-children.

[3] Immigration and Refugee board C. Responses to information requests (RIRs). Sri Lanka Sex Domest violence, Incl Legis state Prot Serv available Vict. 2012;1–8.

[4] Perera B. Prevalence and correlates of sexual abuse reported by late adolescent school children in Sri Lanka. 2009;21(2):203–11. 10.1515/ijamh.2009.21.2.203 19702200

[5] Rajapaksa-hewageegana N, Piercy H, Salway S, Samarage S. Sexual & reproductive healthcare sexual and reproductive knowledge, attitudes and behaviours in a school going population of Sri Lankan adolescents. Sex Reprod Healthc [Internet]. 2015;6(1):3–8. 10.1016/j.srhc.2014.08.001. 10.1016/j.srhc.2014.08.001 25637417

[6] Udigwe I, Ofiaeli O, Ebenebe J, Nri-Ezedi C, Ofora V, Nwaneli E (2021). Sexual abuse among adolescents. Ann Heal Res.

[7] Hettiarachchi D (2022). The place of sexuality education in preventing child pregnancies in Sri Lanka. Sri Lanka J Child Heal.

[8] Downing J, Jones L, Bates G, Sumnall H, Bellis MA. A systematic review of parent and family-based intervention effectiveness on sexual outcomes in young people. Health Educ Res [Internet]. 2011;26(5):808–33. 10.1093/her/cyr019. 10.1093/her/cyr019 21474577

[9] Romo LF, Bravo M, Tschann JM. The effectiveness of a joint mother-daughter sexual health program for Latina early adolescents. J Appl Dev Psychol [Internet]. 2014;35(1):1–9. 10.1016/j.appdev.2013.10.001.

[10] Grusec JE (2011). Socialization processes in the family: social and emotional development. Annu Rev Psychol.

[11] Shams M, Parhizkar S, Mousavizadeh A, Majdpour M. Mothers’ views about sexual health education for their adolescent daughters: a qualitative study. Reprod Health. 2017;14(1). 10.1186/s12978-017-0291-8 PMC5301399 28183332

[12] Mataraarachchi D, Buddhika Mahesh PK, Pathirana TEA, Ariyadasa G, Wijemanne C, Gunatilake I et al. Mother’s perceptions and concerns over sharing sexual and reproductive health information with their adolescent daughters- A qualitative study among mothers of adolescent girls aged 14–19 years in the developing world, Sri Lanka. BMC Womens Health [Internet]. 2023;23(1):223. 10.1186/s12905-023-02369-1. 10.1186/s12905-023-02369-1 PMC10157993 37138289

[13] Rohanachandra YM, Amarakoon L, Alles PS, Amarasekera AU, Mapatunage CN. Parental knowledge and attitudes about child sexual abuse and their practices of sex education in a Sri Lankan setting. Asian J Psychiatr [Internet]. 2023;85:103623. Available from: https://www.sciencedirect.com/science/article/pii/S1876201823001788. 10.1016/j.ajp.2023.103623 37167649

[14] Bessant J, Maher L (2009). Developing radical service innovations in healthcare—the role of design methods. Int J Innov Manag.

[15] Mataraarachchi D, Buddhika APTE, Vithana PKM (2023). Mother-daughter communication of sexual and reproductive health (SRH) matters and associated factors among sinhalese adolescent girls aged 14–19 years, in Sri Lanka. BMC Womens Health.

[16] Pocock SJ. The Size of a Clinical Trial [Internet]. Clinical Trials. 2013. p. 123–41. (Wiley Online Books). 10.1002/9781118793916.ch9.

[17] Robinson WT. Adaptation of the information-motivation-behavioral skills model to needle sharing behaviors and hepatitis C risk: a structural equation model. SAGE Open. 2017;7(1).

[18] John SA, Walsh JL, Weinhardt LS (2017). The information–motivation–behavioral skills Model Revisited: A Network-Perspective Structural equation Model within a Public Sexually Transmitted Infection Clinic Sample of hazardous alcohol users. AIDS Behav.

[19] Eastman KL, Corona R, Schuster MA. Talking parents, healthy teens: a worksite-based program for parents to promote adolescent sexual health. Prevening Chronic Dis Public Heal Res Pract Policy. 2006;3(4). PMC1784238 16978501

[20] Godamunne PKS. Sri Lankan parents’ attitudes towards adolescent reproductive and sexual health education needs: A qualitative study. 2008.

[21] Whalen CK, Henker B, Hollingshead J, Burgess S (1996). Parent–adolescent dialogues about AIDS. J Fam Psychol.

[22] Coombs RH, Santana FO, Fawzy FI. Parent training to prevent adolescent drug use: an educational model. J Drug Issues [Internet]. 1984;14(2):393–402. 10.1177/002204268401400214.

[23] Kirby D, Miller BC. Interventions designed to promote parent-teen communication about sexuality. 2002;(97):93–110. 10.1002/cd.52 14964946

[24] Mytton J, Ingram J, Manns S, Thomas J, Manns S, Hons L et al. Facilitators and barriers to engagement in parenting progrmas. Health Educ Behav. 2014;(May 2013). 10.1177/1090198113485755 23640123

[25] Winick C, Rothacker DQ, Norman RL (2002). Four worksite weight loss programs with high-stress occupations using a meal replacement product. Occup Med (Lond).

[26] Bogart LM, Skinner D, Thurston IB, Toefy Y, Klein DJ, Hu CH (2013). Let’s talk! A South African worksite-based HIV prevention parenting program. J Adolesc Health.

[27] Schuster MA, Corona R, Elliott MN, Kanouse DE, Eastman KL, Zhou AJ (2008). Evaluation of talking parents, Healthy Teens, a new worksite based parenting programme to promote parent-adolescent communication about sexual health: Randomised controlled trial. BMJ.

[28] Akers AY, Holland CL, Bost J (2011). Interventions to improve parental communication about sex: a systematic review. Pediatrics.

