# Supplementary material for: Development and implementation of a worksite-based intervention to improve mothers’ knowledge, attitudes, and skills in sharing information with their adolescent daughters on preventing sexual violence: lessons learned in a developing setting, Sri Lanka
Source: BMC Public Health. 2024 Apr 8;24:983. doi: 10.1186/s12889-024-18416-x (PMC11000366; doi:10.1186/s12889-024-18416-x)
Supplement: Supplementary file 1 — Supplementary Material 1 [file 12889_2024_18416_MOESM1_ESM.docx]

**Annexure 1**

Findings of the descriptive cross-sectional study among adolescent girls and the qualitative study among mother of adolescent girls in Kalutara district (present study setting) that were used to develop the intervention

**Findings of Descriptive study among adolescent girls**

1. Adolescent girls had a close relationship with their mothers compared to fathers. (51%- closest feeling parent was mother. While 44% were close to both).
2. Nearly 90% of the girls were sharing their day to day information with their mothers, every day or most of the days.
3. Over one fourth of the adolescent girls worried that they are often being criticized when having a conversation with their mothers (28%).
4. Nearly 70% of the girls wanted to discuss sex related topics with their mothers.
5. 87% thought discussing sex related matters with mothers will help to protect themselves from future harm
6. Mother was an existing mode of sexual information to >85% of the adolescent girls and nearly 80% preferred to get sex information from mother
7. More than 30% of the mothers refrained from discussing certain topics such as sex and marriage with their adolescent daughters
8. More than 25% were uncertain about their mothers’ ability to respond to their sexual health issues.

**Findings of qualitative study among mothers of adolescent girls**

1. Adolescent girls had a close relationship with their mothers compared to fathers. (51%- closest feeling parent was mother. While 44% were close to both).
2. Nearly 90% of the girls were sharing their day to day information with their mothers, every day or most of the days.
3. Over one fourth of the adolescent girls worried that they are often being criticized when having a conversation with their mothers (28%).
4. Nearly 70% of the girls wanted to discuss sex related topics with their mothers.
5. 87% thought discussing sex related matters with mothers will help to protect themselves from future harm
6. Mother was an existing mode of sexual information to >85% of the adolescent girls and nearly 80% preferred to get sex information from mother
7. More than 30% of the mothers refrained from discussing certain topics such as sex and marriage with their adolescent daughters
8. More than 25% were uncertain about their mothers’ ability to respond to their sexual health issues.
9. Many mothers were against providing sexual health information and family planning information to their children
10. Many mothers thought that medical officers are the best personal to provide SRH information to adolescents
11. Majority of the mothers agreed that they can provide SRH information to their children if equipped with skills and knowledge.
12. Mothers wanted to know what, when and how to communicate sexual issues with adolescent children
13. Mothers wanted to know about the ways to answer the questions raised by their daughters
14. Mothers wanted to know about the most appropriate techniques to initiate a sexual discussion
15. Nearly 30% of the girls were either not satisfied with their mothers’ answers to their sexual health problems.
16. Majority of the girls thought it’s that their mothers will misunderstand them if they do discuss these topics.
17. Many mothers had talked to their adolescent daughters about issues related to menstruation, prevention of sexual violence, keeping personal boundaries.
18. Cultural stigma, mothers’ busy schedule, mothers’ inability to respond to the questions, mothers’ unwillingness to share sex related information, and poor knowledge on an adolescent girls’ needs as barriers to take up a sex related conversation.
19. Many girls expected their mothers to be more knowledgeable about adolescent sexual health matters (79%).
20. Girls expected their mothers to trust them (91.5%) and ensure privacy and confidentiality of the information shared between them (85%)
